# Source Identification and Health Risk Assessment of Heavy Metals in Soil: A Case Study of Lintancang Plain, Northeast China

**DOI:** 10.3390/ijerph191610259

**Published:** 2022-08-18

**Authors:** Qianru Man, Lijuan Xu, Mingfang Li

**Affiliations:** 1 State Key Laboratory of Geological Processes and Mineral Resources, China University of Geosciences, Beijing 100083, China; 2 Linyi Ecological and Environmental Monitoring Center of Shandong Province, Linyi 276000, China

**Keywords:** soil, heavy metals, enrichment, toxic risks, sources analysis, Lintancang Plain

## Abstract

To investigate the concentration, source, and potential health risk of soil heavy metals (V, Cr, Ni, Cu, Zn, Pb, Hg), this study determined the concentration of these seven metals in 37 soil samples from Linyi City, southeast of Shandong Province, China. The mean concentrations of the investigated heavy metals followed the sequence: Cr (76.2 mg/kg) > V (70.5 mg/kg) > Zn (70.1 mg/kg) > Ni (34.0 mg/kg) > Pb (31.4 mg/kg) > Cu (23.2 mg/kg) > Hg (1.7 mg/kg). The enrichment factor (EF) and geo-accumulation index (I_geo_) indicated an extreme enrichment of Hg (EF > 10, I_geo_ > 4) within the study area, while a slight enrichment of other metals. According to the toxic risk index (TRI), Hg accounted for the strongest soil toxicity (TRI = 8.07, 64.3%). The risk assessment with hazard index (HI) suggested that the health risks of all metals were acceptable, and the HI of adults was generally lower compared with that of the children. In addition, two principal components (PC) calculated by principal component analysis (PCA) were used to identify the sources of these heavy metals, which were 57.73% for PC 1 (Pb, Cr, Zn, Ni, Hg, Cu and V) and 21.63% for PC 2 (Hg, Cu and V), respectively. Moreover, PC 1 was mainly controlled by anthropogenic inputs, while PC 2 was contributed to by natural sources. Combined with the correlation matrix, it was concluded that there were three different sources for all seven heavy metals.

## 1. Introduction

Environmental pollution of heavy metals has increased since 1900 [1]. From then on, rapid urbanization and industrialization actively contributed to the release and abundance of heavy metals in the environment [2,3]. By surveying the national soil contamination, Hu et al. [4] found that there were more than 20 million hectares of polluted land in 2011 [5]. Being nondegradable with a high toxicity and strong mobility, the heavy metals in soil are easily absorbed and enriched by crops [6]. Though some heavy metals, such as copper and zinc, are essential elements for the human body in low concentrations [7], at high concentrations, many heavy metals have adverse health effects, causing danger to human lives through the food chain [8,9]. For example, Pb causes negative effects to the skeletal, enzymatic, nervous, immune, and endocrine systems [10,11]. The toxicity of Cr is responsible for lung cancer, liver and kidney damage, weakened immune systems, and cancers of the respiratory tract caused by DNA damage [12]. Moreover, Hg is considered as the most potent neurotoxicant which damages the brain, kidneys, lungs, and chromosomes, disrupts the central nervous system, and causes negative reproductive effects [13].

Shandong Province is located on the east coast of China, with a long history of agriculture. It has the highest ratio of cultivated land areas and its agricultural added value has been ranked first among all provinces in China for a long time. Linyi City is located in the southeast of Shandong Province, Lintancang Plain, which was known as the “granary”. It is the main grain and vegetable production area and is also an important commodity grain base in Shandong Province. Therefore, the soil safety in Lintancang Plain is crucial; it affects the quality of crops in the whole province and even the whole country, given that the soil pollution will lead to the continuous degradation of biological quality and cause a direct threat to the safety of agricultural products. 

To date, several indices have been widely applied to evaluate the toxicity, ecological risk, and contaminated levels of heavy metals in soil. For example, the potential ecological risk index (PERI) [14] and the hazard index (HI) [15] have been applied to evaluate the risk and toxicity in soil, respectively [16], while the pollution condition evaluations of soil were supported with the geo-accumulation index (I_geo_) [17], pollution index (Pi) [18], the enrichment factor (EF) [19], and Nemerow integrated pollution index (PN) [20]. Because of the unique conditions of Shandong Peninsula and its important agricultural industries–fishery economy, this area has been the focus of previous investigations [21,22,23,24] on heavy metal or soil pollution; however, the soil heavy metal assessment of Linyi City, especially in Lintancang Plain has not been studied.

To effectively evaluate the pollution levels of this study area, a comparative assessment of various heavy metal pollution indices as mentioned above was needed. Thus, the heavy metals of soil samples in Linyi City were analyzed in this study. The I_geo_, EF, TRI, and HI indexes were used to assess the pollution condition and potential risk of heavy metals in the soil of Linyi City. A powerful statistical tool, principal component analysis (PCA), was applied to quantitatively identify the sources of heavy metals [14]. The concentration enrichment level and the toxic risk of heavy metals in soil and their sources were assessed in this study, which was important to find priority measures in some future remediation programs [25].

## 2. Materials and Methods

### 2.1. Study Area

Linyi City is located in the southeast of Shandong Province, China, with a population of 10.624 million people and an area of 17,191.2 km^2^, 34°22′–36°13′ N and 117°24′–119°12′ E crossing of this area, which is the largest and most populous prefecture-level city of Shandong Province [26]. The climate is classified as a warm temperate semi-humid mainland monsoon climate, with the mean temperature between 12.4 and 13.4 °C and mean precipitation between 734.8 and 874.1 mm per year [27,28]. The magmatic rocks of Linyi are widely distributed, from ultrabasic to neutral and felsic rock. There are more than 40 kinds of proven rocks in the whole region, such as limestone, gypsum, quartz sandstone, granite, marble, and so on [29,30]. 

### 2.2. Sampling and Chemical Analysis

**Figure 1:**
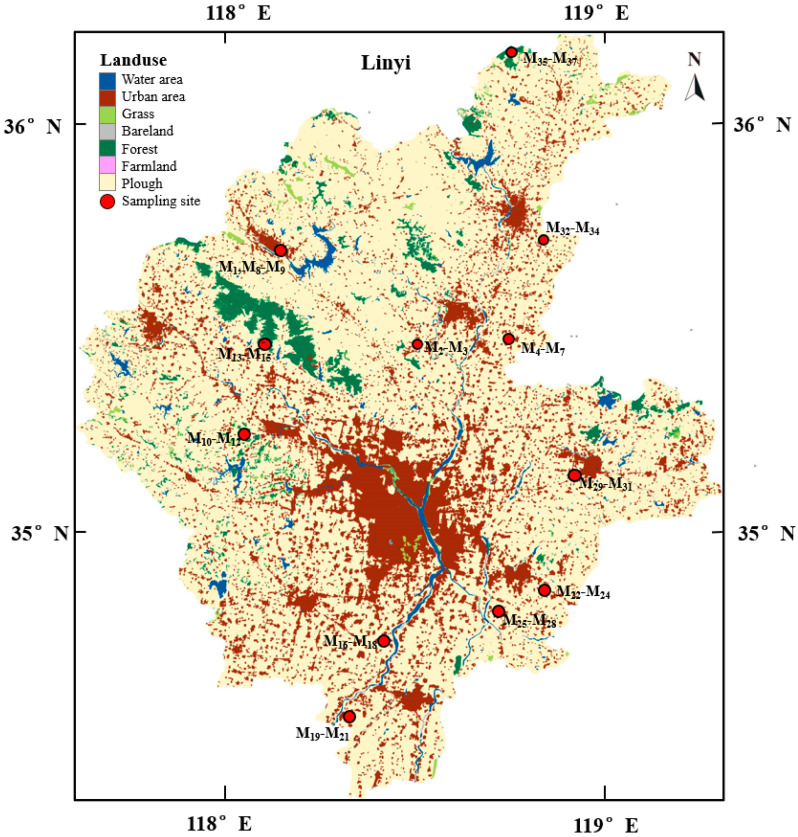
The sample sites and vegetation distributions of Linyi, Shandong Province.

All the field samplings were conducted in June 2018. To prevent the metal shovel’s impact on the trace elements tested, wooden shovels were used. After removing litter from the topsoil, a total of 37 soil samples were collected at a depth of 0–40 cm from sampling locations (Figure 1). It was vital to remove large debris, stones, and pebbles from the soil and put the collected soil into cloth bags and label them. The collected samples were put in a freeze dryer to pre-freeze for 48 h so as to eliminate the moisture content and then they were ground until they could pass through 50 mm sieves. Finally, they were mixed thoroughly to obtain the representative samples and the screened fine soil particles were placed in polyethylene bags for subsequent processing.

The major element (Al, Fe, Na, K, Mg, and Mn) analysis of soil samples was analyzed with the melting method by an X-ray fluorescence spectrometer (XRF) produced by RIGAKU, Japan in Wuhan SampleSolution Analytical Technology Co., Ltd., Wuhan, China. The original data of all major elements was listed in Appendix A (Table A1). The quality control reference materials were GBW07403 and GBW07406, and the X-ray tube was a 4.0 Kw end window Rh target. The test conditions of voltage and current were 50 kV and 60 mA, respectively. All data were corrected by the theoretical α coefficient method and the relative standard deviation (RSD) was better than ±2%. The major element data of GBW07403 and GBW07406 are shown in Table 1.

The trace elements (Cr, Hg, Pb, V, Cu, Zn, and Ni) were measured using a high resolution inductively coupled plasma mass spectrometer (HR-ICP-MS) (Thermo Fisher Scientific, Waltham, MA, USA) in the Isotope Geochemistry Laboratory of China University of Geosciences, Beijing, China. The methods used to digest the soil samples are briefly described as follows: 50 mg of soil powder was digested with 1.5 mL HNO_3_ and 0.7 mL HF in a bomb liner at 175 °C for 48 h (the liner was placed in the steel tank and placed in the oven). Then 1 mL H_2_O_2_ was added to the samples at room temperature at least overnight. After the samples were dried and vaporized at 150 °C on a hot plate, 1 mL HNO_3_ was added, and the bomb liner was put into a steel can and heated in the oven at 175 °C for 24 h. Samples were transferred into the 22 mL beaker, and 4 mL of aqua regia was added after being dried at 130 °C. Then they were heated at 100 °C for 18 h and dried at 150 °C on a hot plate. Finally, 3% HNO_3_ was used to dissolve the remaining digest for a further concentration test. To validate the accuracy, American Bureau of Standard Equipment Laboratory, USA standard solutions Std-1, Std-2 and Std-4 were used. The BHVO-2, BCR-2, and AGV-2 reference materials were measured to assess the data quality (Table 2). Analytical precision for the soil trace elements was better than 10% (1σ) and the accuracy was better than ±5%. The original data of the trace elements analyzed in this study was listed in Appendix A (Table A2).

### 2.3. Appraisal Methods

#### 2.3.1. Geo-Accumulation Index

The geo-accumulation index (I_geo_) was also cited to evaluate the level of heavy metal contamination in the soil, which was established by Müller et al. [31]. It was obtained by comparing the contamination levels between present and former concentrations [32]. This method has been widely used since the late 1960s [31]. The computation of I_geo_ was defined using Equation (1):

(1) $$I_{\mathrm{geo}\;}{=\log}_{2}(\frac{C_{i}}{1{.5\;\times \;B}_{i}})$$

where B_i_ is the local background concentration of metal i (mg/kg) and C_i_ is the concentration of the investigative heavy metal i in the soil samples (mg/kg). The coefficient 1.5 in the equation was used to minimize the effect of some variations in the background values. All reference values of studied metals in Equation (1) were taken from Wei et al. [33]. The I_geo_ for each metal was classified using seven (0–6) enrichment grades [31] listed in Table 3.

#### 2.3.2. Enrichment Factor

In order to assess the enrichment degree of heavy metals in the soils, the Enrichment Factor (EF) was used. In the previous study, Al was defined as a reference element due to its scarcity in various pollution sources and wide distribution in continental rocks [35]. The concentration of heavy metal was normalized to a conservative element by the EF using Equation (2) [36,37,38,39]:

(2) $$\mathrm{EF}=\frac{{{(C}_{i}{/C}_{\mathrm{ref}})}_{\mathrm{soil}}}{{{(C}_{i}{/C}_{\mathrm{ref}})}_{\mathrm{background}}}$$

where (C_i_/C_ref_)_soil_ is the ratio of the concentration of a given metal i to that of Al in a soil sample, and (C_i_/C_ref_)_background_ is the soil background ratio between that metal and Al in Linyi City [33]. The corresponding categorizations of the EF are shown in Table 3.

#### 2.3.3. Risk Assessment

The toxic risk index (TRI) was used to evaluate the integrated toxic risks based on both the probable effect level (PEL) and the threshold effect level (TEL) [40]. This index was applied for ecological risks caused by Cr, Cu, Ni, and Zn [37]. The TRI of the soil was calculated using Equation (3):

(3) $$\mathrm{TRI}=\overset{n}{\underset{i=1}{\sum }}{\mathrm{TRI}}_{i}=\sqrt{\frac{{\left(C_{s}^{i}/C_{\mathrm{PEL}}^{i}\right)}^{2}+{\left(C_{s}^{i}/C_{\mathrm{TEL}}^{i}\right)}^{2}}{2}}$$

where the $C_{s}^{i}$ is the concentration of metal i (mg/kg) in the soil sample, and $C_{\mathrm{PEL}}^{i}$ and $C_{\mathrm{TEL}}^{i}$ are the PEL and TEL of metal i (mg/kg) [41], respectively. There were five categories classified based on the values of TRI (Table 3).

The health risk assessment includes the relationship between the dose and negative health effects and the estimation of the amounts of pollutants entering the body [42,43]. According to the U.S. Agency for Toxic Substances and Disease Registry (ATSDR), Cr and Ni were considered as carcinogenic factors among all studied heavy metals, while other elements (Cu, Zn, V, Pb, and Hg) could cause chronic poisoning though they were non-carcinogenic substances. Moreover, the non-carcinogenic risk is usually assessed by calculating hazard index (HI) values [42]. The dose gained through the three pathways was calculated using D_i_, which was defined using Equations (4)–(6):

(4) $$D_{\mathrm{ing}}=\frac{C\;\times \;\mathrm{IngR}\;\times \;\mathrm{Ef}\;\times \;\mathrm{ED}\;\times \;10^{-6}}{\mathrm{AT}\;\times \;\mathrm{BW}}$$

(5) $$D_{\mathrm{dermal}}=\frac{C\;\times \;\mathrm{Ef}\;\times \;\mathrm{ED}\;\times \;\mathrm{SA}\;\times \;\mathrm{AF}\;\times \;\mathrm{ABS}\;\times \;10^{-6}}{\mathrm{AT}\;\times \;\mathrm{BW}}$$

(6) $$D_{\mathrm{inh}}=\frac{C\;\times \;\mathrm{InhR}\;\times \;\mathrm{Ef}\;\times \;\mathrm{ED}}{\mathrm{AT}\;\times \;\mathrm{BW}\;\times \;\mathrm{PEF}}$$

where the D_i_ is the daily exposed quantities of selected metals according to direct ingestion through the hand–mouth way (D_ing_), dermal absorption of heavy metals in particles adhered to exposed skin (D_dermal_), and inhalation of re-suspended particles through nose and mouth (D_inh_) [43]. Other parameters are shown in Table 4. The formula of HI was as follows:

(7) $${\mathrm{HQ}}_{i}=\frac{D_{i}}{{\mathrm{RfD}}_{i}}$$

(8) $$\mathrm{HI}=\sum^{\;}{\mathrm{HQ}}_{i}$$

where RfD is the reference dose (mg kg^−1^ day^−1^) (Table 5) and the hazard index (HI) is equal to the sum of HQ_i_ (Equation (8)) [44]. There is a probability that non-carcinogenic risks on human body occur if the value of HI exceeds one, and the chance increases as the value rises [34].

#### 2.3.4. Multivariate Analysis

The statistical approaches including the Pearson correlation coefficient and principal component analysis (PCA) were used to obtain descriptive statistics and explore the possible sources of the heavy metals [34]. The correlation coefficients are statistical indicators which are used to reflect the degree of correlation between variables. The PCA was the most popular multivariate statistical tool used to investigate the origins and associations of heavy metals proposed by Hotelling in 1933 [45]. It compressed the dimensionality of the dataset to several influencing factors and succeeded in preserving the relationships presented in the original data [46,47], which could explain the variance and research multivariate relationship of the data.

## 3. Results and Discussion

### 3.1. Concentration of Heavy Metals in Soil

The descriptive statistics of heavy metals of soil are provided in Table 6. The Shapiro–Wilk (S-W) test was used to evaluate the normal distribution of the small size data (*n* = 37). The *p* values of the metals Cr, Ni, Cu, Zn, Pb, and Hg were all less than 0.05, so there were enough reasons to reject the normal distribution of them. Hence, the median concentrations of heavy metals instead of arithmetic means were used for HI calculations, and the arithmetical mean values were used for the further comparison of other indexes [48]. The seven heavy metals were ranked by concentration as follows: Cr (76.2 mg/kg) > V (70.5 mg/kg) > Zn (70.1 mg/kg) > Ni (34.0 mg/kg) > Pb (31.4 mg/kg) > Cu (23.2 mg/kg) > Hg (1.7 mg/kg). The mean concentration of Cr (215.5 mg/kg) was highest compared to other heavy metals mentioned above. The concentrations of Cu, Cr, Ni, Pb, and Hg in soil were also higher than that of soil background values. Hg concentration was approximately 60 times as that of soil background value. The observation indicated that Hg was the most enriched metal in the study area, while the concentrations of others were similar to soil background values in Linyi City.

On a national scale, as shown in Table 7, compared with the first-tier cities of Beijing, Shanghai, and Guangzhou, the concentration of Zn in Linyi city was much lower, the concentrations of Ni and Hg were higher and the concentrations of other metals were at moderate levels [49,50,51]. The content of all comparative metals in the soil of Lintancang Plain of Linyi City was lower than old industrial cities such as Xi’an and Shenyang [52,53], while the rest of the metals were at higher levels than neighboring provinces/cities (Hebei, Jiangsu, and Tianjin), except Cu and Zn [54,55,56].

### 3.2. Geo-Accumulation Index (I_geo_)

**Figure 2:**
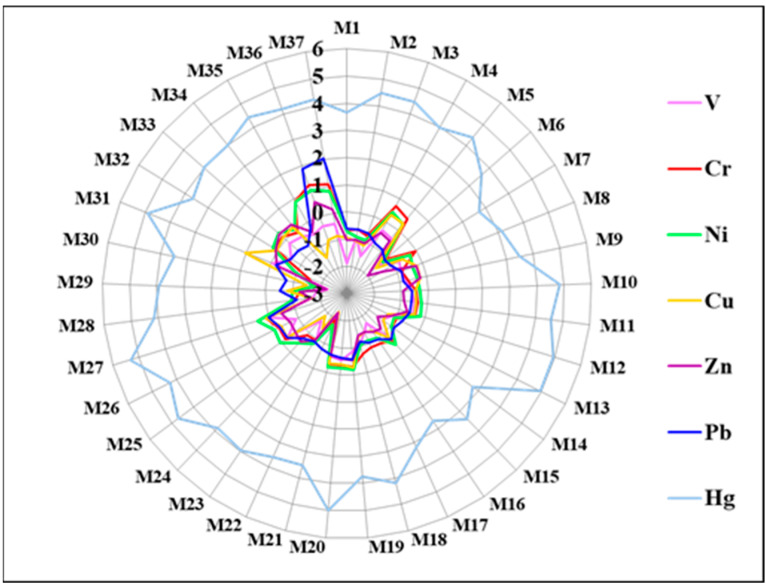
Geo-accumulation index (I_geo_) of selected metals in the soil.

The mean values of I_geo_ decreased in the order of Hg (4.01) > Ni (−0.40) > Cr (−0.45) > Pb (−0.55) > Cu (−0.73) > Zn (−0.82) > V (−0.92) (Figure 2). With a mean I_geo_ value of 4.01, Hg was the most serious contamination heavy metal, and it revealed a heavily polluted level. The mean values of I_geo_ for other metals (Ni, Cr, Pb, Cu, Zn, and V) were less than 0, indicating an unpolluted level. However, besides Hg, the sites M4 and M5 (Yinan County) were lightly polluted with respect to Cr, Ni, and Cu (0 < I_geo_ <1). The mean values of I_geo_ for Cr and Pb in the sites of M36 and M37 were greater than 1, suggesting that Yishui County was moderately polluted with regard to Cr and Pb.

### 3.3. Enrichment Factor

**Figure 3:**
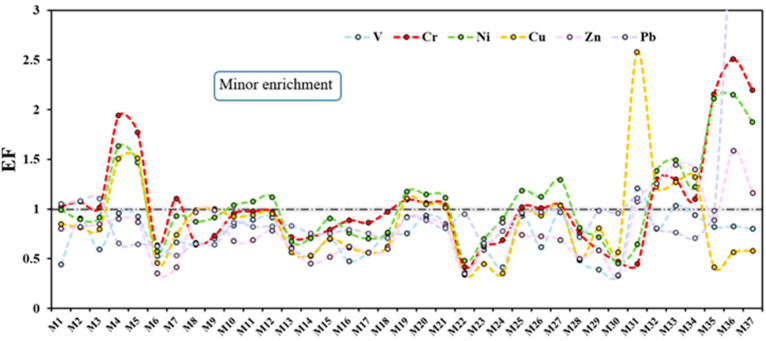
The EF values of each sampling of soil in Linyi City.

The EF values of all metal elements in the 37 samples and the corresponding enrichment level categorizations of EF values are shown in Figure 3 and Table 3, respectively. The mean EF values of all samples showed the same order as that of the I_geo_ index. They declined in the order of Hg (23.17) > Ni (1.05) > Cr (1.04) > Pb (1.01) > Cu (0.87) > Zn (0.79) > V (0.72). Overall, according to EF, Hg could be defined as severe enrichment (10 < EF < 25). Ni, Cr, and Pb indicated a minor enrichment (EF > 1) of these metals, while the residual metals (Cu, Zn, and V) showed no enrichment characteristics in most sampling sites (EF < 1). 

In detail, the EF of Hg ranged from 7.43 to 43.25 and there were 12 sampling points (about a third of all samples) whose values were above 25, which was considered very severe enrichment. It was noted that Hg, without its value in Figure 3, was not of the same order of magnitude as the values of other metals. Similar to that of the I_geo_, the enrichment degrees of Cr, Ni, and Pb in Yishui County (M32-M37) and the Cr, Ni, and Cu in Yinan County (M4 and M5) were all slightly high, which may have been caused by human activities. 

**Figure 4:**
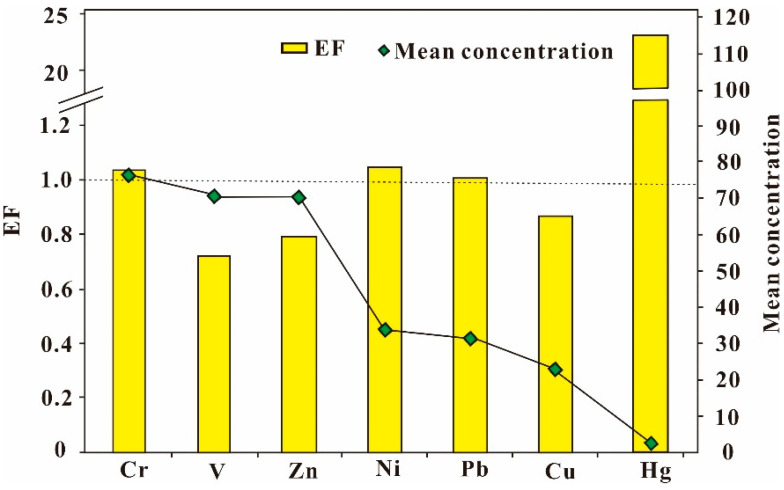
The relationship between EF and the mean concentration of each metal.

Besides, the plot between the mean concentrations of the heavy metals and EF is shown in Figure 4. Hg had the lowest mean value, but the largest EF (>20) compared to other elements. This observation revealed that excess Hg may be attributed to the anthropogenic inputs [66]. A previous study found that Hg was a typical gas-migration element and Shandong Province was a large coal-burning province, especially in winter [67]. The element Hg that was released from coal was transported to the ground through dry and wet deposition [68]. In this way, it could cause the accumulation of this element in soil, which may be the reason why Hg was highly enriched in the soil of Linyi City, Shandong Province.

Instead, even though V had a high mean concentration, it had the lowest EF value among all metals and was not enriched in the soil. Hence, we concluded that V was derived from the parent rocks and was not particularly relevant to human activities [69]. The Pearson correlation coefficients and PCA analysis were further used to identify the sources of these metals.

### 3.4. Potential Risk Assessment

#### 3.4.1. Toxic Risk Index (TRI)

**Figure 5:**
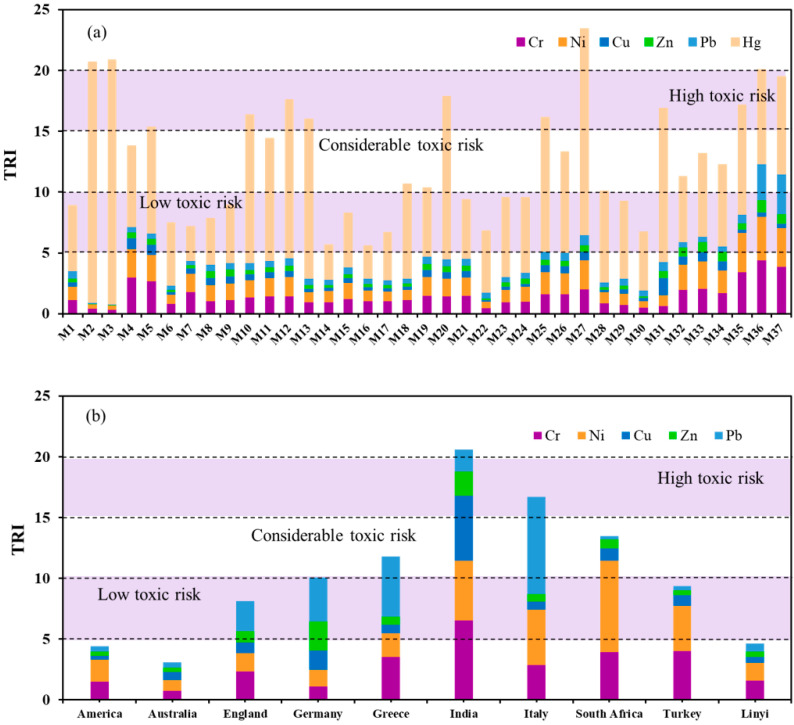
The toxic risk index (TRI) of heavy metals in the soil, (**a**) Linyi City, (**b**) Linyi City with other countries in the world.

As shown in Figure 5a, the values of the TRI of all 37 samples ranged from 5.57 (M16) to 23.40 (M27). Four sites (M2, M3, M27, M36, TRI > 20) presented a high toxic risk, nine sites (M5, M10, M12, M13, M20, M25, M31, M35, and M37, 15 < TRI < 20) presented a considerable toxic risk, while other sites had a moderate toxic risk or a low toxic risk. Different from the order of EF and I_geo_ values, the mean TRI values of individual metals were arranged in this descending sequence of Hg (8.07) > Cr (1.52) > Ni (1.46) > Pb (0.64) > Cu (0.45) > Zn (0.42), with the mean contribution of 64.3%, 12.1%, 11.6%, 5.1%, 3.6%, and 3.3%, respectively. The significant contribution of Hg to the TRI was related to its low TEL, low PEL, and high concentration. This phenomenon highlighted the potential toxicity of soil in Linyi City, especially for Hg.

Comparing the TRI value with different countries of known elements all over the world (Figure 5b), the toxic risk of five metals in the soil of Linyi City was slightly higher than that of America and Australia [57,58], while it was significantly lower than the rest of the countries, especially than those of Italy and India, whose TRI values excessed 15 [59,60]. Beyond that, as for the high mean contribution metals, the data indicated that the TRI contribution values from Cr and Ni in Linyi City merely exceeded those of Australia and Germany [61]. In conclusion, both the TRI and mean concentrations of these heavy metals in Linyi City were relatively low among the surveyed cities in China and other countries around the world, indicating that the potential toxic risk in our study area was at a low level [62,63,64,65]. 

#### 3.4.2. Health Risk Assessment

The mean HI values for adults and children decreased in the order of Cr > Pb > Hg > Ni > Cu > Zn. The computing results are shown in Table 8. The HI value of each element was below 1 among the two groups, indicating that few hazards were presented through the assumed exposure pathways for these metals. In general, the HI values of children in different elements were 3 to 7 times as that of adults, reflecting that children were vulnerable to face greater detrimental health risks. A previous study found that if HI values were greater than 0.1 in the child cohort, adverse human health effects might occur [70]. Consequently, the high HI value (0.377) observed for Cr was an important non-carcinogenic risk factor for children in the study area; therefore, it was vital to pay attention to this element. Considering Cr could also lead to asphyxia by means of reducing oxygen demand of the biochemical process, it sounded a pertinent alarm for environmental safety in the soil of Lintancang Plain.

### 3.5. Sources Analysis

#### 3.5.1. Correlation Analysis Results

Pearson correlation coefficients of the soil heavy metals are shown in Table 9. A significantly positive correlation (*p* < 0.01) combined with the highest correlation coefficient (R^2^ = 0.959) was found between Ni and Cr, suggesting an identical source among them. Cr, Zn, and Pb exhibited remarkably positive correlations (*p* < 0.01) between each other with high correlation coefficients (R^2^ > 0.7), implying that these elements had similar sources [69]. In addition, it should be noted that Cu had a high correlation coefficient (R^2^ > 0.7) with V at the 0.01 significant level. Hence, they may share the same source, which was essential for further PCA analysis. Moreover, for the elements Hg, V, and Hg were correlated at the 0.01 significance level. To a weaker degree, Hg and Cu were correlated at the 0.05 significance level. Considering the lower correlation between Hg and Cr, Ni, Zn, and Pb, the element Hg may have similar sources with Cu and V [43], and they came from different origins with Cr, Ni, Zn, and Pb [69].

#### 3.5.2. Principal Correlation Analysis 

**Figure 6:**
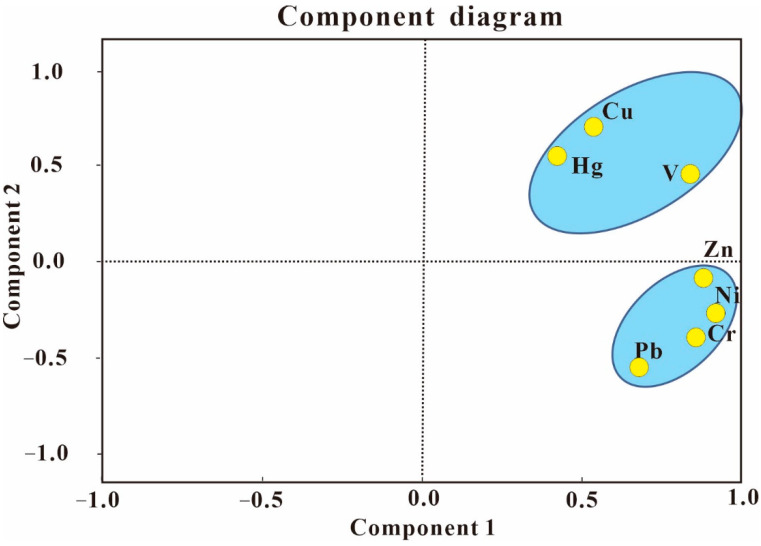
Two-dimensional plot of scores for heavy metals obtained from the PCA results of soil.

According to the PCA coefficients, seven trace metals were divided into two groups, which were the first principal component (PC 1) and the second principal component (PC 2) (Figure 6). They explained 79.4% of the total variance with eigenvalues >1 (Table 10). In previous studies, if the coefficient was greater than 0.7, there were significant or strong loading values [71]. The PC 1 presented the greatest variance (57.7%), having strong positive loadings on Cr, Ni, Zn, and V (>0.7), and moderate positive loading on Pb, Cu, and Hg (0.4–0.7). PC 1 indicated that these heavy metals that were somewhat enriched could be attributed to anthropogenic effects from human activities in the investigated area [69,72,73].

The PC 2 accounted for 21.6% of the total variance and the origins of these heavy metals including V, Cu, and Hg, whose sources were different from those metals in PC 1. It was concluded that they may be from natural sources [74]. In addition, Cu, V, and Hg were distributed in the two components, implying mixed (natural and anthropogenic) origins in the studied area [62]. However, according to the relationship between EF and the concentration of V, high correlation coefficients between V and Cu (0.757), as well as their low EF and low I_geo_ values, it was indicated that V and Cu ultimately came from natural sources with the limited contribution of anthropogenic origins [75,76,77].

In conclusion, the pollution sources of metals Cr, Ni, Pb, and Zn were anthropogenic and Cu and V were from natural sources such as the weathering of parent rocks [69]. The metal Hg was affected by the combination of anthropogenic and natural sources. Similarly, the enrichment factor and correlation analyses results accorded with the PCA analysis.

## 4. Conclusions

Seven heavy metals in soil samples collected from Linyi City, Shandong Province were investigated by statistical techniques, including geo-accumulation index (I_geo_), enrichment factor (EF), toxic risk index (TRI), and hazard index (HI). The concentrations of Cr, Ni, Pb, and Hg in soil were higher than that of the soil background values. According to the EF and I_geo_ values of each element, the concentration degree ranks of seven metals between these two parameters was consistent (Hg > Ni > Cr > Pb > Cu > Zn > V). Hg had a high enrichment level and other elements were slightly or not enriched. Based on the correlation analysis and PCA, Cu and V arose from natural sources due to weathering processes, originating from parent materials. The elements Cr, Ni, Pb, and Zn were derived from anthropogenic input, and Hg was from inputs of the combination of anthropogenic and natural sources. As for the risk evaluation, points with strong toxic risks, individual high-risk metals and their respective contribution rates were analyzed. Generally, for all the metals, few hazards were presented through the three exposure pathways in the Linyi City of Lintancang Plain, China. The potential toxic risk of soil in Linyi City was at a lower level compared with that of other countries of the word. The enrichment condition, pollution level of these seven heavy metals, as well as their toxic risks and sources in soil of Linyi City was assessed, which is essential for the subsequent survey and pollution control.

## Figures and Tables

**Table 1:** The trace element data of the reference materials analyzed in this study.

Sample	Rock Type	V (ug/g)	Cr (ug/g)	Ni (ug/g)	Cu (ug/g)	Zn (ug/g)	Pb (ug/g)	Hg (ug/g)
BHVO-2	basalt	317	283	120	129	104	1.5	0.0023
BCR-2	basalt	419	15.2	12.7	19.1	131	10.6	0.0012
AGV-2	andesite	118	15	19	51.2	87.4	13.5	—

**Table 2:** The major element data of the reference materials analyzed in this study.

Sample	Sample Type	SiO_2_	TiO_2_	Al_2_O_3_	TFe_2_O_3_	MnO	MgO	CaO	Na_2_O	K_2_O	P_2_O_5_	LOI	SUM
		Mass%	Mass%	Mass%	Mass%	Mass%	Mass%	Mass%	Mass%	Mass%	Mass%	Mass%	Mass%
GBW07403	soil	74.82	0.37	12.35	1.99	0.04	0.59	1.26	2.69	3.03	0.08	2.53	99.76
GBW07406	soil	57.03	0.72	21.13	8.02	0.19	0.32	0.21	0.18	1.71	0.07	9.71	99.28

**Table 3:** Contamination and toxic risk categorizations based on the geo-accumulation index (I_geo_), enrichment factor (EF), and toxic risk index (TRI).

I_geo_	Pollution Intensity	EF	Enrichment Level	TRI	Toxic Risk
<0	unpolluted	<1	no enrichment	<5	no toxic risk
0–1	lightly polluted	1–3	minor enrichment	5–10	low toxic risk
1–2	moderately polluted	3–5	moderate enrichment	10–15	moderate toxic risk
2–3	Moderately to heavily polluted	5–10	moderately severe enrichment	15–20	considerable toxic risk
3–4	heavily polluted	10–25	severe enrichment		
4–5	heavily to extremely polluted	25–50	very severe enrichment		

Note: The data are from [34].

**Table 4:** Exposure factors used in the health risk assessment.

Parameter	Description	Unit	Children	Adults
Ef	Exposure frequency	mg kg^−1^	350	350
ED	Exposure duration	day year^−1^	6	30
AT	Average time	day	365 × ED	365 × ED
BW	Average body weight	kg	15	55.9
SA	Exposed skin area	cm^2^	1800	5000
AF	Adherence factor	mg cm^−2^ day^−1^	1	1
ABS	Dermal absorption factor	—	0.001	0.001
PEF	Particle emission factor	m^3^ kg^−1^	1.32 × 10^9^	1.32 × 10^9^
IngR	Ingestion rate	mg day^−1^	200	100
InhR	Inhalation rate	m^3^ day^−1^	5	20

Note: The data taken of exposure factors of soil metal are from [34,43]; —, data are not available.

**Table 5:** Reference dose (mg/kg/day) for different heavy metals.

	Cr	Ni	Cu	Zn	Pb	Hg
RfD_ing_	3 × 10^−3^	2 × 10^−2^	4 × 10^−2^	0.3	3.5 × 10^−3^	3 × 10^−4^
RfD_inh_	2.86 × 10^−5^	2.06 × 10^−2^	4.02 × 10^−2^	0.3	3.52 × 10^−3^	8.57 × 10^−5^
RfD_dermal_	6 × 10^−5^	5.4 × 10^−3^	1.2 × 10^−2^	0.06	5.25 × 10^−4^	2.1 × 10^−5^

Note: RfD_ing_, the values of reference dose according to direct ingestion through the hand–mouth way [43]; RfD_inh_, the values of reference dose by means of inhalation of re-suspended particles through the mouth and nose [43]; RfD_dermal_, the values of reference dose according to dermal absorption of heavy metals in particles adhered to exposed skin [43].

**Table 6:** The heavy metal contents (mg/kg) and descriptive statistics in soil of Linyi City (*n* = 37), and the local soil background values.

Parameter	V	Cr	Ni	Cu	Zn	Pb	Hg
Median	72.5	60.9	31.4	20.0	69.8	24.6	1.5
Range	25.8–124.9	23.7–215.5	11.7–81.5	7.0–69.3	23.7–166.2	17.4–160.1	0.6–3.9
BG	82.4	61	26.9	22.6	74.2	26	0.065
AM	70.5	76.2	34.0	23.2	70.1	31.4	1.7
SD	26.0	43.3	16.7	12.2	32.4	29.1	0.8
TEL	—	37.3	18	35.7	123	35	0.174
PEL	—	90	36	197	315	91.3	0.486
S-W test	0.403	0.000	0.002	0.001	0.019	0.000	0.046

Note: Units in mg kg^−1^ for heavy metals; BG, soil background values of Linyi City [33]; AM, arithmetical mean; SD, arithmetical standard deviation; TEL, threshold effect level; PEL, probable effect level; the values of TEL and PEL of heavy metals were inferred from [41]; S-W test, the Shapiro–Wilk test.

**Table 7:** Comparison of soil metal concentrations in Linyi City with those in Chinese cities and other countries in the world.

	V	Cr	Ni	Cu	Zn	Pb	Hg
This study	70.5	76.2	34	23.2	70.1	31.4	1.7
Beijing	—	63.57	27.12	35.49	145.68	36.43	0.87
Guangzhou	—	22.4	11.1	11	277	65.4	—
Shanghai	—	87.72	—	27.8	99.36	28.86	—
Tianjin	—	70.7	27.9	24.3	79.1	11.9	—
Jiangsu	—	76	32.9	26	73	26.8	0.082
Hebei	—	60.4	27.03	22.84	67.71	19.83	0.058
Xi’an	—	167.28	—	94.98	421.46	230.52	—
Shenyang	—	67.9	—	41.6	234.9	80.2	—
Australia	—	36.11	20.56	32.56	58.74	20.28	—
England	80	113	35	43	147	116	—
Germany	—	53	31	81	381	168	0.6
India	—	317.74	112.05	265.87	323.125	83.2	—
America	64	73	41	15	58	20	0.03
Turkey	—	194.73	85.02	43.19	65.1	17.01	—
South Africa	—	191.94	170.63	50.07	123.91	11.87	—
Italy	—	139.33	103.33	33	100.67	371	—
Greece	—	171.325	44.6	34.275	108.625	229.025	—

Note: Data of soil metal concentrations in other areas are from [49,50,51,52,53,54,55,56,57,58,59,60,61,62,63,64,65]; —, no data.

**Table 8:** The calculated HQ values for three exposure routes and HI values in this study.

	Cr	Ni	Cu	Zn	Pb	Hg
HQ_ing_-c	0.260	0.020	0.006	0.003	0.090	0.064
HQ_ing_-a	0.035	0.003	8.58 × 10^−4^	3.99 × 10^−4^	0.012	0.009
HQ_inh_-c	5.16 × 10^−4^	3.69 × 10^−7^	1.20 × 10^−7^	5.63 × 10^−8^	1.69 × 10^−6^	4.24 × 10^−6^
HQ_inh_-a	5.53 × 10^−4^	3.96 × 10^−7^	1.29 × 10^−7^	6.05 × 10^−8^	1.82 × 10^−6^	4.55 × 10^−6^
HQ_dermal_-c	0.117	6.69× 10^−4^	1.91 × 10^−4^	1.34 × 10^−4^	5.39 × 10^−3^	8.22 × 10^−3^
HQ_dermal_-a	0.087	4.99 × 10^−4^	1.43 × 10^−4^	9.98 × 10^−5^	4.02 × 10^−3^	6.13 × 10^−3^
HI-c	0.377	0.021	0.007	0.003	0.095	0.072
HI-a	0.122	0.003	0.001	0.0005	0.016	0.015

Note: HQ_ing_-c, the calculated values of HQ_ing_ for children; HQ_ing_-a, the calculated values of HQ_ing_ for adults; HQ_inh_-c, the calculated values of HQ_inh_ for children; HQ_inh_-a, the calculated values of HQ_inh_ for adults; HQ_dermal_-c, the calculated values of HQ_dermal_ for children; HQ_dermal_-a, the calculated values of HQ_dermal_ for adults; HI-c, the HI values of children; HI-a, the HI values of adults.

**Table 9:** Pearson correlation coefficients for the soil metals.

Element	V	Cr	Ni	Cu	Zn	Pb	Hg
V	1						
Cr	0.584 **	1					
Ni	0.700 **	0.959 **	1				
Cu	0.757 **	0.181	0.286	1			
Zn	0.703 **	0.702 **	0.788 **	0.514 **	1		
Pb	0.306	0.716 **	0.657 **	−0.022	0.580 **	1	
Hg	0.532 **	0.146	0.239	0.341 *	0.168	0.127	1

Note: * Statistically significant coefficients at the *p* < 0.05 level. ** Statistically significant coefficients at the *p* < 0.01 level.

**Table 10:** Initial eigenvalue, rotation sums of squared loadings, and the principal component of each metal.

Component	Initial Eigenvalue			Rotation Sums of Squared Loadings			Element	Principal Component	
	Total	% of Variance	Cumulative%	Total	% of Variance	Cumulative%		1	2
1	4.041	57.732	57.732	4.041	**57.732**	57.732	Ni	0.928	−0.251
2	1.514	21.63	79.361	1.514	**21.63**	79.361	Zn	0.882	−0.046
3	0.783	11.191	90.553				Cr	0.871	−0.390
4	0.359	5.124	95.676				V	0.858	**0.439**
5	0.193	2.751	98.427				Pb	0.673	−0.548
6	0.09	1.288	99.716				Cu	0.541	**0.710**
7	0.02	0.284	100				Hg	0.403	**0.547**

## Data Availability

The data presented in this study are available upon request from the corresponding author.

## Appendix A

**Table A1:** The original data of all major elements.

Sample	SiO_2_	TiO_2_	Al_2_O_3_	TFe_2_O_3_	MnO	MgO	CaO	Na_2_O	K_2_O	P_2_O_5_	LOI	SUM
M1	73.11	0.80	12.19	4.36	0.06	1.05	0.71	1.34	2.16	0.07	3.87	99.71
M2	73.95	0.83	12.04	4.13	0.06	1.07	0.72	1.35	2.15	0.06	4.02	100.38
M3	74.53	0.85	11.51	3.71	0.05	1.00	0.66	1.30	2.11	0.06	3.88	99.66
M4	60.63	0.84	16.37	6.41	0.10	2.34	2.19	2.59	2.12	0.36	5.81	99.76
M5	61.16	0.82	16.32	6.11	0.10	2.12	2.24	2.61	2.20	0.34	5.49	99.49
M6	70.42	0.68	14.42	3.28	0.07	0.75	2.22	2.99	2.66	0.07	2.51	100.07
M7	63.46	0.77	17.71	5.57	0.06	0.93	1.76	2.26	2.34	0.06	4.66	99.57
M8	58.85	0.76	17.15	5.65	0.06	2.31	2.89	3.41	2.27	0.19	6.06	99.59
M9	60.45	0.78	16.88	5.47	0.06	2.16	2.99	3.40	2.30	0.19	4.63	99.31
M10	66.13	0.81	15.31	5.98	0.13	1.34	1.09	0.91	2.58	0.10	5.44	99.81
M11	66.12	0.81	15.75	6.06	0.13	1.35	0.97	0.83	2.72	0.07	5.05	99.85
M12	65.62	0.79	15.89	6.04	0.11	1.52	0.88	0.91	2.82	0.08	4.63	99.28
M13	68.10	0.51	14.25	3.93	0.07	0.92	2.19	2.48	3.13	0.16	3.62	99.37
M14	68.44	0.51	14.56	4.21	0.08	0.91	2.10	2.30	3.08	0.08	3.07	99.34
M15	64.51	0.60	16.64	5.49	0.09	1.19	1.63	1.72	3.05	0.06	4.32	99.29
M16	71.20	0.65	12.82	3.88	0.06	1.13	1.95	2.62	2.41	0.15	2.57	99.46
M17	71.29	0.65	13.05	3.94	0.08	1.17	2.11	2.67	2.42	0.13	2.31	99.82
M18	72.28	0.67	12.51	3.78	0.07	1.11	1.95	2.58	2.35	0.11	2.15	99.55
M19	65.18	0.80	14.75	5.72	0.10	1.71	1.48	1.61	2.56	0.12	5.23	99.28
M20	65.95	0.82	14.54	5.60	0.10	1.73	1.53	1.66	2.56	0.13	5.04	99.65
M21	65.25	0.83	15.28	6.04	0.09	1.77	1.47	1.54	2.63	0.09	5.12	100.11
M22	76.27	0.48	12.23	1.82	0.14	0.45	0.65	3.40	2.83	0.12	2.05	100.42
M23	66.20	0.59	16.48	4.78	0.06	1.18	0.68	2.92	3.04	0.04	3.64	99.62
M24	67.39	0.54	15.65	4.11	0.04	1.60	0.61	3.79	3.31	0.18	2.49	99.69
M25	61.95	0.92	17.23	6.47	0.10	1.43	1.54	1.53	2.28	0.09	6.66	100.20
M26	61.57	0.92	17.28	6.47	0.10	1.44	1.49	1.53	2.32	0.09	6.51	99.70
M27	54.89	0.88	20.69	7.53	0.12	1.88	1.84	0.87	1.98	0.10	9.05	99.83
M28	57.61	0.56	12.58	3.29	0.08	1.32	9.56	2.05	2.28	0.07	9.89	99.29
M29	69.26	0.74	14.05	3.98	0.09	0.87	1.26	2.77	3.27	0.19	3.27	99.76
M30	73.83	0.58	12.63	2.57	0.07	0.53	0.84	2.82	3.54	0.07	2.09	99.57
M31	59.26	1.04	15.75	7.63	0.15	2.09	2.61	3.31	3.62	0.61	3.59	99.63
M32	58.91	1.01	16.52	8.84	0.11	1.94	2.19	2.66	2.70	0.38	4.56	99.82
M33	57.05	1.03	17.00	9.65	0.13	2.07	2.73	2.76	2.70	0.52	4.09	99.72
M34	58.05	1.02	16.79	9.10	0.11	1.85	2.64	2.79	2.90	0.50	3.77	99.51
M35	56.26	0.71	17.10	7.32	0.09	3.61	2.70	3.31	1.39	0.11	7.00	99.58
M36	54.09	0.64	18.67	8.59	0.08	3.77	2.83	3.23	1.45	0.06	6.10	99.51
M37	55.32	0.61	18.90	8.21	0.07	3.26	2.86	3.26	1.26	0.06	5.82	99.62

**Table A2:** The original data of the trace elements analyzed in this study.

Sample	V	Cr	Ni	Cu	Zn	Pb	Hg
M1	33.50	57.56	24.54	17.68	54.62	25.24	1.24
M2	67.80	59.55	21.89	16.72	55.30	25.54	2.16
M3	42.56	53.82	21.45	15.63	54.67	24.95	2.18
M4	97.70	146.17	54.23	42.06	82.90	21.01	1.53
M5	93.98	132.92	48.65	42.01	79.32	20.70	2.02
M6	47.18	42.30	16.85	11.21	28.34	17.78	1.19
M7	73.23	89.93	33.40	22.32	41.27	18.50	0.65
M8	68.39	52.03	30.37	28.89	93.08	21.89	0.87
M9	73.70	56.90	31.42	28.77	93.22	21.26	1.09
M10	79.42	67.33	32.34	24.13	58.08	25.90	2.82
M11	87.30	70.94	34.44	25.26	60.77	25.38	2.32
M12	90.57	71.22	36.09	25.80	69.78	25.83	3.02
M13	54.69	47.07	19.52	13.79	48.19	23.30	3.03
M14	47.77	48.18	20.90	13.20	36.97	21.59	0.65
M15	72.51	60.86	30.58	19.97	48.39	24.59	1.03
M16	37.86	52.37	19.78	13.44	44.85	19.99	0.61
M17	45.45	51.80	18.60	12.48	41.17	19.28	0.90
M18	58.22	56.16	19.46	13.31	41.87	17.39	1.79
M19	69.43	74.32	35.23	27.88	76.15	26.54	1.31
M20	84.72	71.10	33.94	25.97	73.37	25.32	3.09
M21	80.92	72.96	34.65	26.43	70.51	24.14	1.12
M22	27.43	23.66	11.94	7.05	23.89	22.80	1.17
M23	60.48	47.35	23.31	12.63	58.57	21.27	1.51
M24	40.30	49.62	28.79	9.42	75.56	23.95	1.42
M25	99.67	80.89	41.42	28.09	71.45	32.52	2.54
M26	66.56	80.21	39.45	27.61	70.14	32.86	1.91
M27	124.86	99.10	54.44	36.48	80.01	39.38	3.92
M28	38.02	43.78	20.74	10.48	35.38	17.66	1.73
M29	34.01	37.90	20.40	19.36	45.92	27.03	1.47
M30	25.76	27.41	11.66	12.22	23.73	23.71	1.10
M31	118.55	32.41	20.68	69.32	95.17	34.27	2.92
M32	82.26	98.70	46.41	34.47	116.13	26.20	1.24
M33	109.42	101.60	51.58	36.83	137.68	25.50	1.57
M34	98.36	84.96	41.72	37.91	131.45	23.29	1.54
M35	87.15	169.56	73.34	12.10	84.85	32.94	2.07
M36	96.18	215.48	81.49	17.97	166.18	142.33	1.79
M37	94.18	191.08	71.89	18.72	123.18	160.10	1.85

Note: Units in mg kg^−1^ for trace metals.
